# A Practical Method for Red-Edge Band Reconstruction for Landsat Image by Synergizing Sentinel-2 Data with Machine Learning Regression Algorithms

**DOI:** 10.3390/s25113570

**Published:** 2025-06-05

**Authors:** Yuan Zhang, Zhekui Fan, Wenjia Yan, Chentian Ge, Huasheng Sun

**Affiliations:** 1 School of Geographic Sciences, East China Normal University, Shanghai 200241, China; yzhang@geo.ecnu.edu.cn (Y.Z.); 51263901051@stu.ecnu.edu.cn (Z.F.); 51263901085@stu.ecnu.edu.cn (C.G.); 2 Shanghai Information Technology Research Center, Shanghai 200125, China; wj.yan@outlook.com; 3 Shandong Provincial Key Laboratory of Soil and Water Conservation and Environmental Protection, School of Resources and Environment, Linyi University, Linyi 276000, China

**Keywords:** Landsat red edge, Landsat OLI, Sentinel-2 MSI, spectral consistency, red-edge reconstruction, red-edge indices

## Abstract

Red-edge bands are the most essential spectral data for multispectral remote sensing images, with them playing a critical role in monitoring vegetation growth status at regional and global scales. However, the absence of red-edge bands limits the applicability of Landsat images, the most widely used remote sensing data, to vegetation monitoring. This study proposes an innovative method to reconstruct Landsat’s red-edge bands. The consistency in corresponding bands of Landsat OLI and Sentinel-2 MSI was first investigated using different resampling approaches and atmospheric correction algorithms. Three machine learning algorithms (ridge regression, gradient boosted regression tree (GBRT), and random forest regression) were then employed to build the red-edge reconstruction model for different vegetation types. With the optimal model, three red-edge bands of Landsat OLI were subsequently obtained in alignment with their derived vegetation indices. Our results showed that bilinear interpolation resampling, in combination with the LaSRC atmospheric correction algorithm, achieved high consistency between the matching bands of OLI and MSI (*R*^2^ > 0.88). With the GBRT algorithm, three simulated OLI red-edge bands were highly consistent with those of MSI, with an *R*^2^ > 0.96 and an *RMSE* < 0.0122. The derived Landsat red-edge indices coincide with those of Sentinel-2, with an *R*^2^ of 0.78 to 0.95 and an *rRMSE* of 3.37% to 21.64%. This study illustrates that the proposed red-edge reconstruction method can extend the spectral domain of Landsat OLI and enhance its applicability in global vegetation remote sensing. Meanwhile, it provides potential insight into historical Landsat TM/ETM+ data enhancement for improving time-series vegetation monitoring.

## 1. Introduction

Since the 1970s, the Landsat series of satellites have continuously observed the world, for a period of over 50 years. They have acquired a large amount of high-quality remote sensing data for terrestrial surface monitoring and have made an unprecedented contribution to global resource and environmental applications [1]. The Landsat-9 satellite was successfully launched on 27 September 2021, which dramatically promoted the satellite’s capability to repeatedly observe the Earth [2]. This allows people to acquire remote sensing images covering most regions of the planet at time intervals of about eight days [3]. Landsat remote sensing data have been used in various studies, such as those on vegetation, soil, hydrology, ecology, and many others [4,5,6]. In vegetation remote sensing, Landsat data are extensively used in mapping covers and change detection, forest monitoring, crop area mapping and yield estimation, and wetland vegetation and grassland monitoring [7,8,9,10]. Moreover, the time-series vegetation index derived from historical Landsat images has also become an important data source for phenology detection in recent years [11,12]. It can be used for not only monitoring vegetation growth and health status with high accuracy [13] but also for identifying phenological features for various vegetation types [14,15]. However, one of the most significant shortcomings of Landsat data is the lack of red-edge spectral information, which dramatically limits the application of Landsat data in vegetation growth monitoring and health status diagnosis [16].

The red edge of a remote sensing image is the spectral region between the red and near-infrared bands, with electromagnetic wavelengths between 700 to 800 nm. This region exhibits a large, sharp rise due to the contrast between the strong absorption of chlorophyll and the high internal leaf scattering of vegetation canopies [17]. The red edge has been proven to be an ideal spectral range for species identification and biophysical/biochemical parameter inversion [18,19,20,21]. This spectral region has also proven to be the most sensitive to disease symptoms and could serve as a leading indicator when it comes to detecting plant stress early [22]. This has led to an increasing number of satellites being sent to space to carry sensors that are sensitive to these wavelengths. Moreover, there is an increasing number of studies that deal with the classification of healthy and infested plants based on spectral signatures in the red-edge spectral region.

Currently, multispectral satellite images containing the red-edge band mainly include RapidEye, WorldView-2/3, Sentinel-2, and GaoFen-6. Compared with traditional multispectral sensors, those with red-edge bands have significant advantages in various applications. For land use/cover mapping, Sentinel-2 multispectral images with red-edge bands also show higher classification accuracy than those without red-edge bands, such as Landsat [23]. For estimating the leaf area index of crops using RapidEye data, the vegetation index with red-edge information can increase accuracy by 10% [24]. The fusion of RapidEye and Sentinel-2 data dramatically improves the accuracy of grassland mapping, which provides vital information for the sustainable management of pastures [25]. When WorldView-2/3 is used to accurately classify agriculture, forestry, and urban tree species, the red-edge band makes a significant contribution [26,27]. Past studies have confirmed that the red-edge vegetation index derived from Sentinel-2 data can significantly improve the inversion accuracy of vegetation biochemical parameters [28,29]. Recent studies have also proven that the red-edge band of Chinese GaoFen-6 can significantly improve fragmented rice paddy mapping and enhance farmland drought monitoring [30,31]. The normalized difference red-edge index of GaoFen-6 can improve the discrimination accuracy of forest health status from 70% to 90% [32]. In addition, the Triple Red-Edge Index (TREI) of Sentinel-2 is proposed to capture the reflectance curve changes in the red-edge region, which provides a comprehensive measure of vegetation health and growth, making it suitable for crop yield prediction [33]. Red-edge-based vegetation indices were also used for crop yield prediction, yielding a concordance correlation coefficient of for canola (0.89) and wheat (0.85) [34]. The above studies indicate the application value of the red-edge band in vegetation remote sensing monitoring.

The comprehensive applicability of Landsat data allows researchers to make numerous attempts to reconstruct the red-edge band for Landsat data. As an early study, the 15 standard bands of MERIS were used to determine the red-edge position of vegetation canopies and simulate the red-edge index with a linear interpolation method [35]. In recent years, studies on the spectral-domain extension of Landsat multispectral data, especially Landsat red-edge (LRE) band reconstruction, have been attracting more attention. Shamsoddini and Raval (2018) used WorldView-2 data to predict the red edge of Landsat-5 via multiple linear regression, and they found that the derived red edge-based vegetation indices performed better in mature native Eucalyptus forest than those without a red-edge band [36]. However, this study did not quantify the impacts of the satellite sensors’ variation and data acquisition conditions. Scheffler et al. (2020) oversampled the bandwidth of airborne APEX and HyMap hyperspectral images to simulate the spectral characteristics of OLI and MSI data based on the spectral response function [37]. The simulated data were used to train a multivariate linear regression model for extending the spectral domain of OLI to MSI. However, when the model was applied to satellite images, various factors caused unstable errors. Mukherjee and Liu (2023) utilized a conditional generative adversarial network (GAN) to predict the first red edge (Band5 with a central wavelength of 705 nm) of Sentinel-2 MSI by using the green band (Band3, 525–600 nm) of Landsat-8 OLI, and two other red-edge bands (MSI Band6, 740 nm, and Band7, 783 nm) by the near-infrared band (OLI Band5, 845–885 nm) [38]. The results showed that the limited spectral information of a single band inevitably resulted in undesirable prediction accuracy. Also, GAN model training is challenging due to its complex structure. In general, the above studies on the reconstruction of the LRE band are based on highly consistent non-homologous data. However, less attention is paid to the unique spectral features of different land surfaces.

Since the spectral response curve of Landsat OLI is very similar to that of Sentinel-2 MSI, the comparability between the two satellites’ data is attracting more and more attention [39,40,41]. The correlation between the band information of remote sensing images has demonstrated the feasibility of LRE band reconstruction by synergizing the corresponding bands of the OLI and MSI [42]. Therefore, our study objectives are to quantitatively analyze the impact of various data processing chains on the expansion of the spectral domain and to develop a practical cross-sensor synergy method for Landsat red-edge reconstruction. This study will substantially contribute to the following: (1) the enhancement of Landsat OLI spectral information; (2) the generation of a Landsat red-edge vegetation index for promoting global vegetation growth monitoring, plant nutrient and health status diagnosis, and biochemical parameter quantification; and (3) the improvement of new scientific insights into future cross-sensor synergy of spaceborne multispectral data. Generally, our proposed method is expected to substantially expand the application domain of Landsat multispectral remote sensing data in future sustainable resource management and risk assessment of the Earth’s environment.

## 2. Materials and Methodology

### 2.1. Remote Sensing Data

The Landsat-8 satellite, equipped with an operational land imager (OLI) and a thermal infrared sensor (TIRS), was launched in February 2013. It captures global surface information with a swath width of 185 km and a 16-day revisit cycle. The OLI includes nine reflective bands, i.e., blue, green, red, and near-infrared, and two shortwave infrared bands with a spatial resolution of 30 m for land applications. To ensure continuity in Earth surface observation, Landsat-9, as a sister satellite of Landsat-8, carrying a payload of OLI-2 and TIRS-2, was launched in September 2021. This allows the two satellites to collect highly consistent earth surface information, enabling the acquisition of remote sensing images for any global region every eight days at most [3]. Landsat imagery with the highest data quality through rigorous terrain and atmospheric correction is available for free from the U.S. Geological Survey (USGS) protocol EarthExplorer (https://earthexplorer.usgs.gov/ (accessed on 10 October 2024)).

Being supported by the Copernicus program of the European Space Agency (ESA), Sentinel-2A, equipped with multiple spectral instruments (MSIs), was launched in September 2015. It features a larger swath width of 290 km at a shorter revisit period of 10 days [43]. To further improve the observation frequency, Sentinel-2B was launched in March 2017, officially forming the Sentinel-2 constellation with Sentinel-2A, enhancing the temporal resolution to 5 days at most. MSI data have thirteen reflectance bands, including four visible and near-infrared (VNIR) bands with 10 m spatial resolution, six bands at 20 m for vegetation red-edge, near-infrared, and shortwave infrared, and three 60 m bands [43]. Like USGS, ESA also provides global users with high-quality Sentinel-2 MSI data free of charge via its website (https://dataspace.copernicus.eu/ (accessed on 10 October 2024)).

The Landsat OLI (NASA, USA) and Sentinel-2 MSI (ESA) data used in this study are level-1C (L1C) data products with rigorous terrain corrections and the level-2A (L2A) surface reflectance data through further atmospheric corrections. The L1 and L2 data products of both Landsat OLI and Sentinel-2 MSI record top-of-atmosphere (TOA) reflectance and bottom-of-atmosphere (BOA) reflectance of the Earth’s surface, respectively. Since both Landsat OLI and Sentinel-2 MSI have a high temporal resolution, this makes it possible to observe many places on Earth with identical overpass dates. To ensure the spectral comparability of two remote sensing images, a crucial prerequisite is to ensure that their observation over the target areas takes place on the same day. In addition, it is vital to obtain as clear images as possible to minimize the spectral differences between images acquired by two satellite sensors. Considering different geographical distribution patterns and land cover types, this study selected 11 from 18 pairs of Landsat-8 and Sentinel-2A images to evaluate the spectral correlation in corresponding bands. They were used to construct the red-edge simulation model. The remaining 7 paired images at ROI-2, ROI-8, and ROIs-14 to 18 were used to investigate the validity of the reconstructed LRE bands (Table 1).

### 2.2. Land Surface Reflectance Conversion

Although all of the datasets have been terrain corrected and geocoded, slight geographic discrepancies exist between Sentinel-2 and Landsat L1C and L2A data due to their inconsistent spatial resolution (10 or 20 m for Sentinel-2 bands against 30 m for Landsat) [38]. Therefore, it is necessary to conduct precise positional alignment and pixel resizing for subsequent accurate spectral comparison. Generally, these deviations stem from systematic errors in image pairs from the two datasets [44]. This study employs the blur-invariant phase correlation method to co-register Sentinel-2 and Landsat images [45]. Geometric errors for each pair of images are less than half a pixel (<9.62 m).

To explore the spectral consistency between the two datasets, the nearest-neighbor (NN) and bilinear interpolation (BI) algorithms were separately employed to resample all of the image bands of Sentinel-2 MSI to keep their pixel size consistent with that of the Landsat image (30 m). In addition, any pixels with cloud contamination were investigated using the cloud masks generated by the Fmask algorithm [46]. As presented in Table 1, although the Landsat OLI and Sentinel-2 MSI were acquired on the same day, they did not observe the regions of land surfaces at the same moment, that is to say, with different imaging times. There is a 1 to 25 min time interval for those observation areas spanning different latitude zones. Hence, there could be some pixels in the two remote sensing images with outliers due to variable cloud cover and atmospheric noise [47]. These outliers were excluded by applying an outlier handling method proposed by [48].

**Figure 1:**
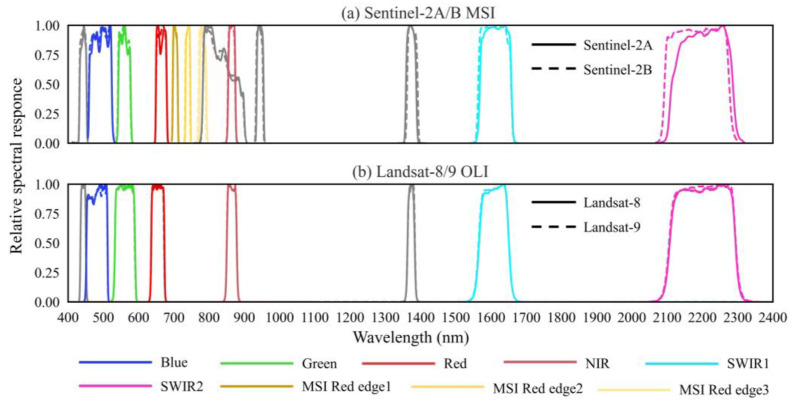
Spectral response for Sentinel-2 MSI and Landsat OLI bands.

Except for the coastal aerosol and cirrus bands, Landsat OLI and Sentinel-2 images have six matched spectral bands with similar central wavelengths (CW) and bandwidths (Table 2). The spectral response features of Sentinel-2 MSI and Landsat OLI also exhibit high similarity (Figure 1). The band parameters of the two sensors were compared to characterize the spectral band specifications. The most considerable difference in CW and bandwidth is less than 10 nm and 20 nm, respectively (Table 2). The consistency of spectral features makes it feasible to reconstruct Landsat OLI through Sentinel-2 MSI band information.

Correlation analysis was applied to quantify the spectral agreement in six matched band pairs of Landsat and Sentinel-2 MSIs, i.e., three visible bands (blue, green, and red), MSI red edge4 and OLI NIR, and two short-wave infrared bands (SWIR1 and SWIR2). Moreover, machine learning-based regression analysis was employed to characterize the interrelation between the three red-edge bands of Sentinel-2 MSI and the six other bands concerning diverse land surface types such as vegetation, water surfaces, and built-up areas. Modeling the interrelation is vital for linking three red-edge bands (with CWs of 705 nm, 740 nm, and 783 nm) with the other six bands of the Sentinel-2 MSI image. The best-fitting model will be transferred to simulate the red-edge bands for Landsat OLI. The simulated red-edge bands were compared with those of Sentinel-2 MSI for accuracy examination.

### 2.3. Red-Edge Prediction Modeling and Performance Evaluation

With the unique spectral features of diverse Earth surface objects, complex nonlinear correlations exist between reflective spectral bands. Moreover, the consistency in the corresponding band settings of the two sensors allows the spectral domain of Landsat OLI data to be expanded for OLI red-edge reconstruction (RER). Three widely used machine learning regression algorithms (i.e., ridge regression (RR), gradient boosted regression tree (GBRT), and random forest regression (RFR)) were selected and used to explore the correlation between three red-edge bands (bands 5, 6, and 7) of Sentinel-2 and the other six bands (bands 2, 3, 4, 8A, 11, and 12). Due to the ability of the RR model to overcome the multicollinearity problem between variables, it performs at a level beyond many traditional multivariate linear regression methods [49]. The GBRT and RF use boosting and bagging learning strategies to better characterize the nonlinear relationship between data [50,51], respectively.

With the RR, GBRT, and RFR algorithms, three regression models were trained by taking the six multispectral bands of the MSI as the input (feature variables) and the three RE bands as the output (target variables), respectively (Table 2). These trained models consequently predicted three simulated RE bands of Landsat OLI, with six original OLI spectral bands serving as inputs.

Sentinel-2 images covering 11 regions of interest (ROIs) were used to train the RR and GBRT models for different land cover types (mainly vegetation), with an allocation ratio of 8:2 for the training and testing datasets. Bayesian optimization was applied to retrieve the parameters of RR and the GBRT to ensure that the regression model could converge to an optimal solution. For RF, a grid-search procedure was employed to search for the appropriate number of trees and the depth value. To balance model performance and efficiency, the number of trees was set to 300, with a depth value of 20.

To evaluate the validity of prediction performance, the simulated LRE reflective bands were compared to the corresponding Sentinel-2 ones. In addition, three commonly used red-edge indices (REIs), the normalized difference red-edge index (NDREI), the chlorophyll index of red-edge (CIre), and the Inverted Red-Edge Chlorophyll Index (IRECI), were derived from the simulated LRE bands (Table 3). Similar to spectral reflectance, these derived Landsat REIs were compared with the Sentinel-2 REIs for consistency evaluation. The coefficient of determination (*R*^2^), root mean square error (*RMSE*), and relative *RMSE* (*rRMSE*) were used to quantify the simulation performance of model training and testing, which are calculated using the following:

(1) $$R^{2}=1-\sum_{i=1}^{n}{\left(y_{i}-{\hat{y}}_{i}\right)}^{2}/\sum_{i=1}^{n}{\left(y_{i}-\bar{y}\right)}^{2}$$

(2) $$RMSE=\sqrt{\sum_{1}^{n}{\left(y_{i}-{\hat{y}}_{i}\right)}^{2}/n}$$

(3) $$rRMSE=RMSE/\bar{y}\cdot 100$$

where *n* is the total number of pixels; $y_{i}$ denotes the reference value of the *i*th pixel; ${\hat{y}}_{i}$ represents the predicted value of the *i*th pixel; and $\bar{y}$ represents the average of all of the reference values.

## 3. Results

### 3.1. Consistency Analysis of OLI and MSI Spectral Bands

#### 3.1.1. Comparison of TOA Reflectance with Different Resampling Algorithms

**Figure 2:**
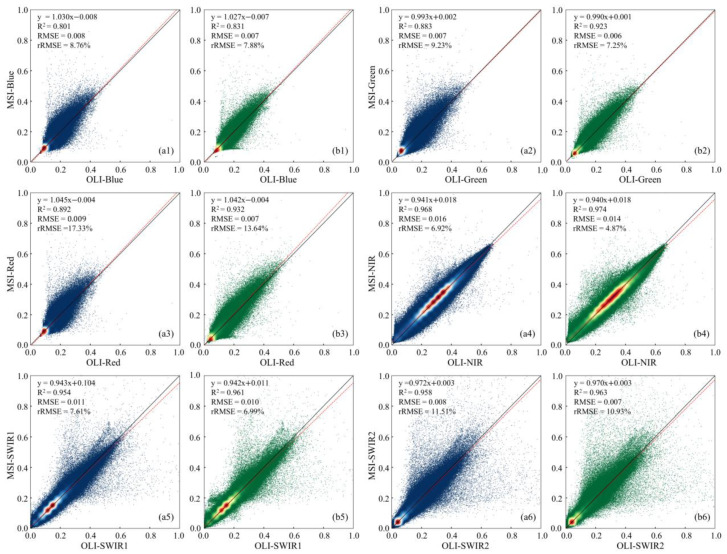
Scatterplots of TOA reflectance in OLI vs. MSI with the nearest neighbor (**a1**–**a6**) and bilinear interpolation (**b1**–**b6**) resampling methods.

Taking Landsat OLI bands as a benchmark, the counterparts of Sentinel-2 MSI were resampled to 30 m of spatial resolution with nearest neighbor (NN) and bilinear interpolation (BI), respectively. In each of the six matching bands in Table 2, the pixel reflectance of the resampled MSI is consistent with that of OLI for both resampling methods, with an *R*^2^ of 0.80 to 0.97 (Figure 2). Compared with NN, BI exhibits better spectral consistency, whereas for the relatively shorter visible spectrum, such as the blue and green bands, the spectral variations between OLI and MSI are observable with an *R*^2^ < 0.9 for NN. This could be attributed to intrinsic differences in OLI and MSI band specifications. On the one hand, there are variations in the spectral response function of the two blue bands (Figure 1). On the other hand, there is an overpass time difference between the two satellites (Table 1). During the time interval of ~20 min, slight changes in the weather or atmospheric environment could cause an observable variation in shortwave TOA reflectance. As a result, the BI method was selected to resample the Sentinel-2 MSI bands for subsequent red-edge reconstruction modeling.

#### 3.1.2. Comparison of BOA Reflectance with Different Atmospheric Correction Algorithms

**Figure 3:**
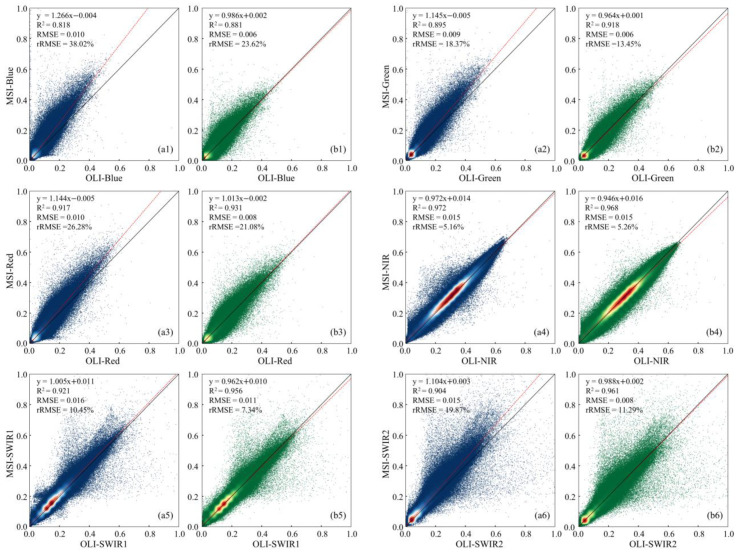
Scatterplots of BOA band reflectance in OLI vs. MSI with Sen2cor (**a1**–**a6**) and LaSRC (**b1**–**b6**) algorithms.

The Sentinel-2 L2A BOA reflectance data product was generated from the L1C data with an atmospheric correction algorithm tool called Sen2cor (https://step.esa.int/main/snap-supported-plugins/sen2cor/ (accessed on 10 October 2024)), whereas that of Landsat OLI L2A was generated with the LaSRC (Land Surface Reflectance Code v3.5.5) algorithm [55]. The L1C data of OLI and MSI were fed into the two algorithms to calculate their reflectance products, respectively. With Sen2cor, a direct comparison of the existing L2A data of the OLI and MSI shows considerable variations in their corresponding bands (Figure 3(a1–a6)). Better agreement between the OLI and MSI was characterized by the NIR (*R*^2^ = 0.972 and *RMSE* = 0.015) and SWIR1 (*R*^2^ = 0.921 and *RMSE* = 0.016) bands, while three visible bands (blue, green, and red) and the second shortwave infrared band (SWIR2) show the most variations relatively. Identity line deviations were the highest in the blue band, with a slope of 1.266 (Figure 3(a1)). Therefore, they could not be applied correctly to LRE reconstruction.

In contrast, LaSRC exhibits more stable consistency in all corresponding bands with an *R*^2^ of 0.881 to 0.968 and an *RMSE* of 0.006 to 0.015 (Figure 3(b1–b6)). The SWIR2 band presents the best fit line with a slope of 0.988 (Figure 3(b6)). Therefore, the LaSRC algorithm was more suitable for generating the surface reflectance data of all spectral bands in the OLI and MSI. They were subsequently used for RER modeling and validation.

### 3.2. The Simulated Red-Edge Bands and Consistency Assessment

In comparison with Sentinel-2 MSI, all three simulated LRE bands show better agreement by using the GBRT with an *R*^2^ > 0.96. The simulated LRE-3 with a central wavelength of 783 nm maintains high consistency with Sentinel-2 MSI band 5, with an *RMSE* < 0.008 and an *R*^2^ > 0.98 (Table 4). However, a relatively larger simulation error was found in LRE-3 (783 nm) for the RR algorithm with an *RMSE* of 0.0132. As a result, the optimal regression model with the GBRT was employed for the RER of Landsat OLI.

**Figure 4:**
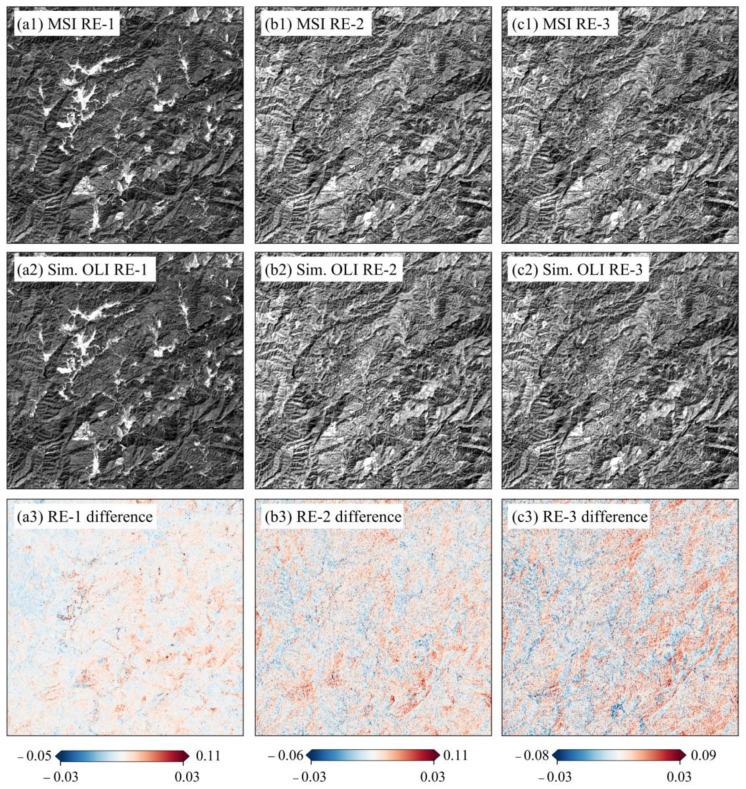
Comparison of three simulated LRE bands with their corresponding MSI ones in the reflectance differences of forestlands.

**Figure 5:**
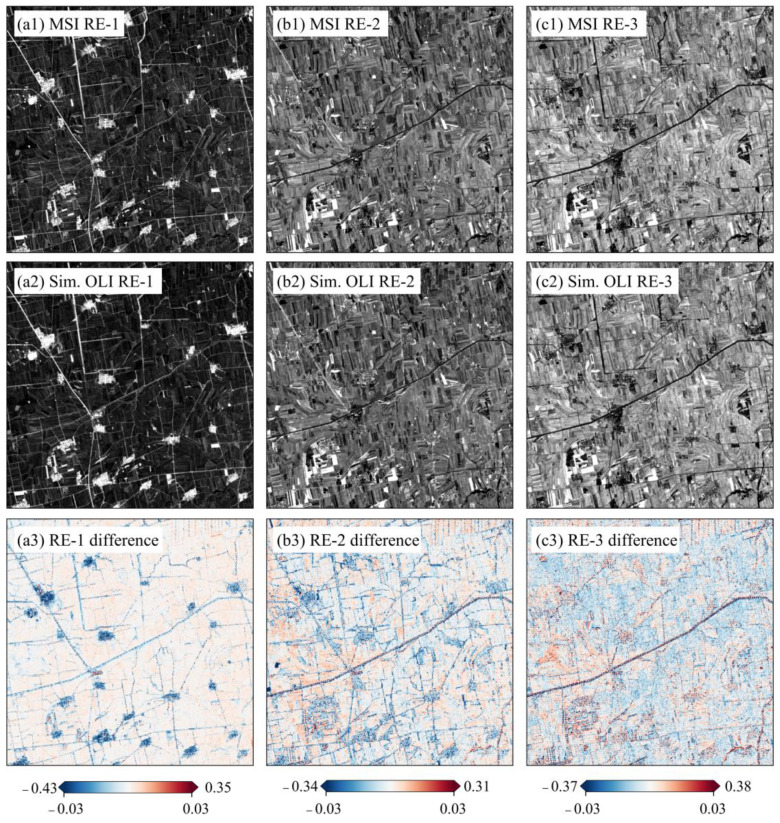
Comparison of three simulated LRE bands with their corresponding MSI ones in the reflectance differences of croplands.

**Figure 6:**
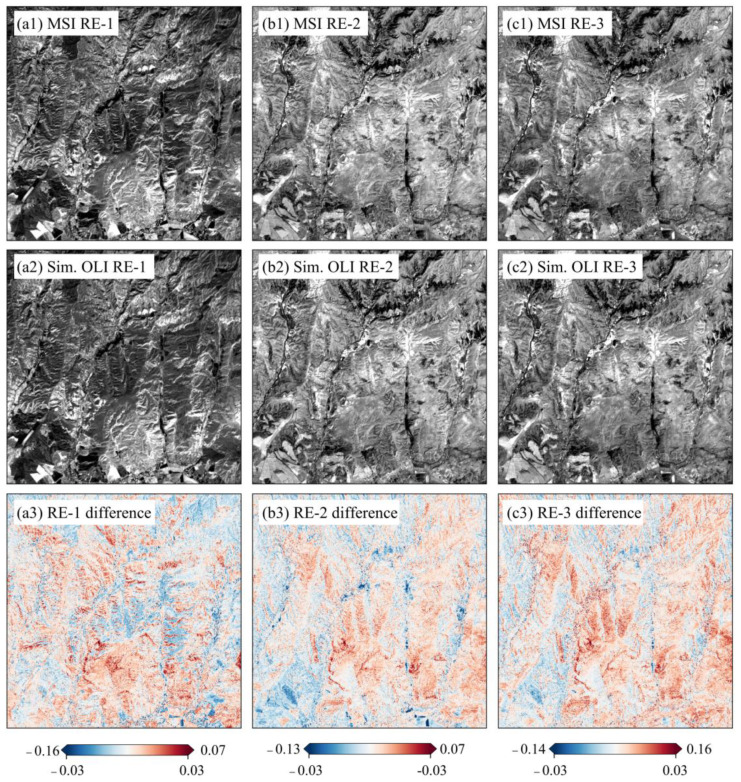
Comparison of three simulated LRE bands with their corresponding MSI ones in the reflectance differences of grasslands.

Three demonstration areas characterized by typical vegetation categories, i.e., crop (ROI-2), forest (ROI-8), and grass (ROI-14), were selected to investigate the validity of the reconstructed LRE bands. These LREs were separately compared to the matching REs of Sentinel-2 MSI band-by-band (Figure 4, Figure 5 and Figure 6). For mountain forestlands, large differences were exhibited in ridgelines and those areas with very steep slopes (Figure 4), which are attributed to significant terrain variation [46]. In plain areas, they mainly distribute in the water surfaces, those areas with shallow vegetation cover, sand-mud flats, and built-up areas (Figure 5 and Figure 6).

For all three vegetation covers, the simulated LRE bands are consistent with those of the MSI, and over 98% of pixels have absolute differences in reflectance of less than 0.03 (Table 5). The highest uniformity is present in the RE-1 of the forest, whereby only 0.01% of pixels have an absolute difference greater than 0.03. Otherwise, the RE-3 of grass exhibits a relatively low one, and the number of pixels with a slightly larger difference accounts for 1.29% of the entire ROI-14.

### 3.3. The REIs Derived from Simulated LRE Bands and Consistency Assessment

**Figure 7:**
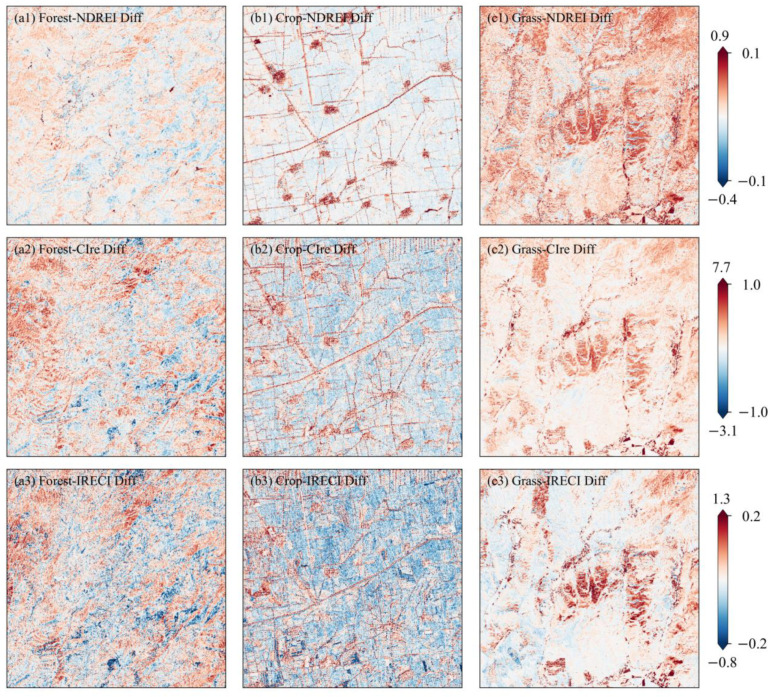
Difference maps of the NDREI, CIre, and IRECI derived from the simulated LRE and Sentinel-2 MSI.

To evaluate the applicability of the reconstructed OLI red-edge bands in vegetation applications, they were used to compute the Landsat REIs, i.e., NDREI, CIre, and IRECI. The difference maps were separately derived by subtracting the simulated Landsat REIs from the Sentinel-2 ones (Figure 7). Through comparison of their spatial distribution, the three Landsat REIs have smaller deviations than those derived from Sentinel-2. Those areas with large discrepancies are mainly found in bare lands, water surfaces, and built-up areas.

The percentage of pixels was calculated from the difference map of three REIs with respective thresholds (Table 6). There are over 98.7% of pixels in the NDREI, with an absolute difference of less than 0.1 for forestlands, croplands, and grasslands, respectively. Similarly, the number of pixels with an IRECI difference of less than 0.2 accounts for over 96% of each land cover. Different from NDREI and IRECI, a threshold of 1.0 was set for CIre difference. Pixels with differences from −1.0 to 1.0 account for over 97% of the ROIs in forestlands, croplands, and grasslands.

**Figure 8:**
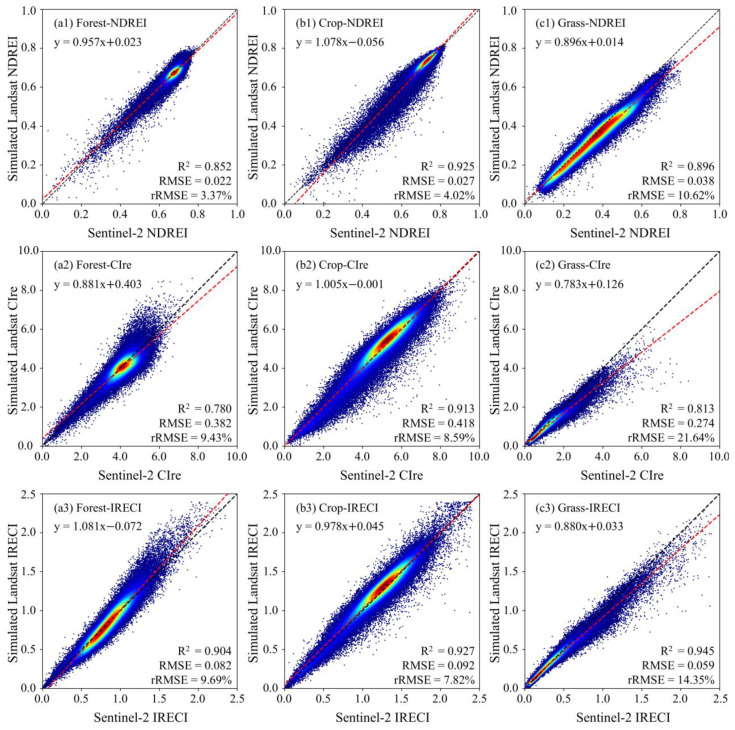
The scatterplots of NDREI, CIre, and IRECI derived from the simulated LRE vs. Sentinel-2 MSI.

As seen in the scatterplots of the three indices (Figure 8), the simulated Landsat NDREI and IRECI exhibit better consistency with those derived from Sentinel-2 than CIre, with an *RMSE* of <0.04 and a relatively smaller *rRMSE* of <15% for all three vegetation types (Figure 8(a1–c1)). As far as the vegetation types are concerned, the simulated Landsat REIs for croplands more closely match the Sentinel-2 ones than those for forestlands and grasslands, with a larger *R*^2^ > 0.913 and a smaller *rRMSE* < 8.6% (Figure 8(b2)). This could be attributed to the larger coverage of croplands. Most crops are in their peak growth period in August, so their dense canopy is less affected by soil background and topographic relief. In contrast, three grass indices show more significant deviations than those of the forest and crop. The grass CIre exhibits the most deviation from the identity line with an *rRMSE* over 20% (Figure 8(c2)). The grasslands have a relatively lower vegetation cover in high latitudes in early May. The evident underestimation of grassland with higher REIs is primarily distributed in those areas with larger surface slopes and two ponds in the central and the bottom right area of ROI-14, respectively (Figure 7(c3)).

## 4. Discussion

Data conversion and complementation between Landsat OLI and Sentinel-2 MSI can substantially enhance their respective applicability [56]. Past studies have been conducted on integrating Landsat OLI and Sentinel-2 MSI data by downscaling the spatial resolution of the MSI images to match OLI bands and applying cross-sensor transformation coefficients through linear fitting [57,58]. In these studies, the applicability of targeted data harmonization models is generally limited to specific study areas because the spectral discrepancies might result from sensor specification, image geometry, weather conditions, and atmospheric correction algorithms [48,59]. In this study, we developed a practicable and reliable spectral reconstruction schema based on the strong correlation between the corresponding bands of the two satellites’ images. The most significant innovation is that an effective Landsat red-edge reconstruction model can be constructed based on Sentinel-2 data alone. This reconstructed model can be transferred directly to Landsat data. This greatly improves the efficiency of Landsat red-band reconstruction. Furthermore, with this proposed method, the Landsat OLI containing reconstructed red-edge bands and the existing Sentinel-2 MSI will complement each other for global vegetation monitoring and environmental management.

The variations in spatial resolution, geographic mismatch, and spectral inconsistency between multisource remote sensing data significantly confine their synergic applications. To minimize these variations, rigorous comparison and rectification are essential for data processing procedures, such as pixel resampling, georeferencing, and atmospheric correction. Existing L2 BOA surface reflectance data products of Sentinel-2 MSI and Landsat OLI cannot be directly combined to apply to vegetation remote sensing due to the significant variations in image resolution and band reflectance. With bilinear interpolation resampling and the LaSRC atmospheric correction algorithm, high spatial and spectral consistency is achievable between the matching bands of the two satellites’ images.

**Figure 9:**
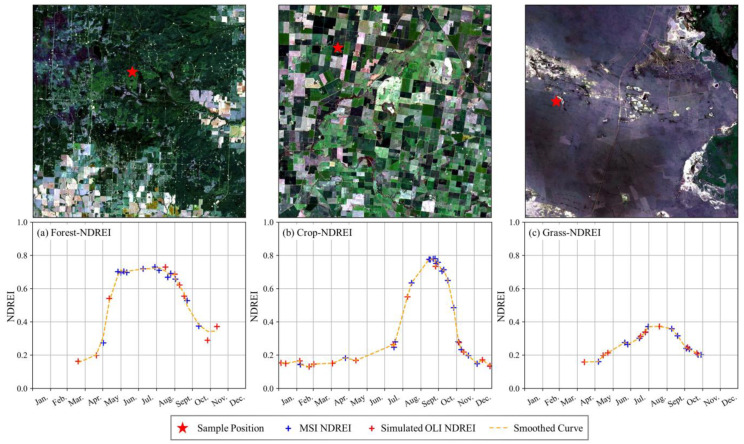
Synergy of Landsat-simulated and Sentinel-2-derived NDREIs for vegetation growth monitoring.

The proposed method not only improves the accuracy of red-edge simulation but also provides a theoretical basis for the cross-sensor simulation of red-edge bands and the derived vegetation indices. As a demonstration study, an attempt was made at three sites at forestland (55.642° N, 119.256° W), cropland (36.440° S, 142.858° E), and grassland (48.556° N, 118.539° E) across the globe (Figure 9). With our proposed RER method, the time-series images of Landsat and Sentinel-2 were acquired to investigate their synergy in the seasonal dynamics of vegetation growth in 2023. The fit lines of the NDREI derived from both reconstructed Landsat and Sentinel-2 data precisely delineate the phenological features of three typical vegetation covers (Figure 9a–c). This implies that the cross-sensor image stack of the reconstructed Landsat OLI and Sentinel-2 MSI is very valuable and desirable in quantifying the physiological properties of vegetation canopies and characterizing their phenological trajectories [39,60,61].

**Figure 10:**
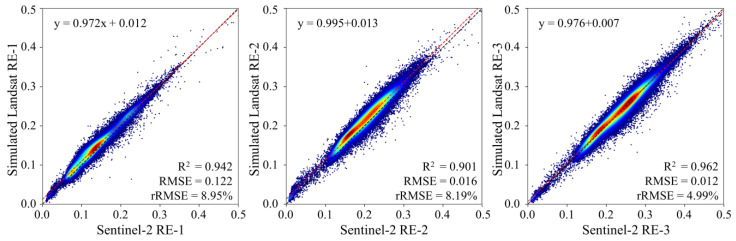
Validation maps for randomly selected validation sites covered by multiple vegetation classes, such as forest, crop, grass, and others.

Four additional validation sites containing more diverse vegetation types were also investigated to further illustrate the practicability of the trained model in our study (see ROIs-15 to -18 in Table 1). We compared all pixels of Landsat OLI and Sentinel-2 MSI images containing forests, crops, and grasses. The total statistical results remain highly consistent for the three simulated red-edge bands (Figure 10), with an *R*^2^ > 0.9 and an *rRMSE* < 9.0%. However, it was found that the statistical errors were slightly larger than those derived from the single vegetation class shown in Table 4. This could be attributed to the limited input for our present model, which was trained from only 11 sites (Table 1). Combining the Sentinel-2 and Landsat missions enables more frequent global vegetation and environmental monitoring with high spatiotemporal variability. For our present reconstruction model, its representativeness, generality, and robustness need to be further improved in future applications. A possible solution might be to extend the spatial extent of data acquisition at global scales and to obtain as many Sentinel-2 images as possible, covering different seasons and vegetation types, to train the GBRT red-edge reconstruction model.

Temporally, the present LRE reconstruction modeling did not take into account the impact of vegetation’s phenological signatures on the variability of spectral features. Most remote sensing images of the two satellites selected in this study were acquired during the intense vegetation period (Table 1), to a certain extent, which might limit the trained model’s applicability to a regional or global scale. This mainly depends on good weather conditions when two satellites synchronously overpass the geographic area. The biophysical and biochemical properties of forests, crops, and grass change significantly throughout the growing season, especially for those at high latitudes [62]. Ideally, given that the two satellites, Landsat and Sentinel-2, can simultaneously acquire data at various development stages and in different growth seasons for diverse vegetation types, it is expected that a red-edge simulation model can be obtained with more general applicability and better performance.

Otherwise, the generation of a general and robust RER model for a global application depends on substantial support with a high-performance computation facility. RER model training based on a powerful cloud platform like GEE, AWS, and Aliyun would be an optimal alternative to deal with such an enormous workload. As reported by the authors of [22], the addition of red-edge bands to Landsat data is expected to greatly enhance application potential in improving land use/cover mapping and change detection. More importantly, the Landsat data archive was announced as open to all users worldwide in 2008. This makes all archived and planning Landsat satellite image data available for free, with it becoming a more valuable data source for future monitoring of Earth’s resources and environment [4]. Thus, long-term historical Landsat data, such as TM/ETM+ images, need to be fully exploited and utilized to further enhance their application value.

## 5. Conclusions

In this study, we propose a cross-sensor spectral reconstruction scheme for Landsat OLI multispectral images in view of the red edge’s importance for vegetation remote sensing monitoring. Many pairs of Landsat OLI and Sentinel-2 MSI images acquired on the same observation days were substantially compared band by band (i.e., blue, green, red, NIR, SWIR1, and SWIR2) with three common vegetation types (crop, forest, and grass). Robust regression models established from six corresponding bands (B2, B3, B4, B8A, B11, and B12) and three red-edge bands (B5, B6, and B7) of MSI images were utilized to simulate Landsat OLI red edges. The primary findings of this study are as follows: (1) With bilinear interpolation resampling and the LaSRC atmospheric correction algorithm, high spatial and spectral consistency is achievable between those matching bands of Landsat OLI and Sentinel-2 MSI images. (2) Comparative analysis indicates that GRBT performs better in modeling the nonlinear correlation between multispectral bands of Sentinel-2 MSI. The regression model is well suited for cross-sensor red-edge band reconstruction for the currently operating Landsat OLI. (3) Accuracy assessment of the simulated Landsat red-edge vegetation indices further demonstrates the feasibility of red-edge band reconstruction of Landsat OLI for the efficient characterization of vegetation type, structure, and condition.

In total, the Sentinel-2 MIS, with comparable bands to Landsat OLI, holds promising synergistic potential to extend the spectral information of Landsat data. What is a more positive prospect is that the third Copernicus Sentinel-2 satellite (Sentinel-2C) was successfully launched on 5 September 2024. Like its siblings, Sentinel-2A and Sentinel-2B, it will continue to provide continuous imagery of the Earth in 13 spectral bands with diverse resolutions. Just recently, the satellite was put into the commissioning phase and began the routine acquisition of high-resolution images of the Earth’s surface. In the near future, we will acquire more quality data from Sentinel-2 MSI to train a more robust large model to reconstruct the red edges of Landsat OLI, even for long-term historical data such as Landsat-7 ETM+ and Landsat-5 TM. This would provide an extensive perspective and new insights into quantifying and monitoring vegetation health and ecosystem functioning at regional and global scales.

## Figures and Tables

**Table 1:** The acquisition information on Landsat OLI and Sentinel-2 MSI imagery covers the same regions of interest (ROIs).

ROI	Acquisition Date (dd/mm/yy)	Central Location (Latitude, Longitude)	Imaging Time (hh:mm:ss)		Dominant Land Covers
			Landsat-8	Sentinel-2A	
1	6 August 2018	(47.440° N, 131.150° E)	10:01:26	10:16:01	Crop
2	6 August 2018	(47.265° N, 132.209° E)	10:01:26	10:16:01	Crop
3	21 September 2023	(37.507° N, 116.414° E)	10:54:23	10:55:31	Crop
4	4 September 2020	(33.802° N, 114.086° E)	10:55:41	11:05:49	Crop
5	10 May 2022	(37.839° S, 142.443° E)	10:15:42	10:20:49	Crop
6	7 December 2021	(27.782° N, 117.672° E)	10:38:55	10:51:11	Forest
7	16 October 2023	(25.724° N, 114.376° E)	10:51:57	10:57:09	Forest
8	16 October 2023	(24.778°N, 113.565° E)	10:51:33	10:57:09	Forest
9	31 August 2023	(46.154° N, 87.629° W)	10:33:53	10:48:51	Forest
10	9 September 2023	(55.544° N, 119.001° W)	10:53:18	11:09:11	Forest and grass
11	9 September 2022	(46.515° N, 129.484° E)	10:08:51	10:15:39	Forest and crop
12	9 September 2022	(41.773° N, 128.527° E)	10:10:26	10:15:39	Grass and forest
13	21 June 2022	(48.788°N,118.519° E)	10:57:04	11:05:51	Grass
14	18 May 2024	(44.084° N, 81.310° E)	10:19:46	10:26:49	Grass
15	1 May 2023	(52.808° N, 24.599° E)	11:18:46	11:30:31	Forest, crop, and grass
16	14 August 2023	(40.023° N, 44.863° E)	10:37:42	10:46:19	Forest, crop, and grass
17	30 September 2023	(39.130° N, 91.554° W)	10:48:22	10:51:11	Forest, crop, and grass
18	25 October 2024	(54.240° N, 113.522° W)	10:28:59	10:53:59	Forest, crop, and grass

**Table 2:** Parameters for the selected bands of Sentinel-2 MSI and Landsat OLI.

Band Name		CW (nm)		Bandwidth (nm)		Spatial Resolution (m)	
MSI	OLI	MSI	OLI	MSI	OLI	MSI	OLI
B2: Blue	B2: Blue	490	482	457–522	450–515	10	30
B3: Green	B2: Green	560	563	543–578	525–600	10	30
B4: Red	B4: Red	665	655	650–680	630–680	10	30
B5: Red edge1	-	705	-	698–713	-	20	30
B6: Red edge2	-	740	-	732–747	-	20	30
B7: Red edge3	-	783	-	773–793	-	20	30
B8a: Red edge4	B5: NIR	865	865	855–875	845–885	20	30
B11: SWIR1	B6: SWIR1	1610	1605	1565–1655	1560–1651	20	30
B12: SWIR2	B7: SWIR2	2190	2200	2100–2280	2100–2300	20	30

**Table 3:** The vegetation indices relating to red-edge bands.

Red-Edge Index	Formula	References
NDREI	(*RE1* − *NIR*)/(*RE1* + *NIR*)	[52]
CIre	*NIR/RE1* − 1	[53]
IRECI	(*RE3 − R*)/(*RE1/RE2*)	[54]

**Table 4:** Consistency assessment of simulated red-edge bands of Landsat against those of Sentinel-2.

Algorithm	Statistical Measures	RE-1 (705 nm)	RE-2 (740 nm)	RE-3 (783 nm)
RR	*R* ^2^	0.9771	0.9483	0.9735
	*RMSE*	0.0082	0.0132	0.0130
	*rRMSE*	9.57%	6.22%	4.84%
GBRT	*R* ^2^	0.9807	0.9651	0.9764
	*RMSE*	0.0076	0.0108	0.0122
	*rRMSE*	8.86%	5.09%	4.54%
RFR	*R* ^2^	0.9739	0.9646	0.9764
	*RMSE*	0.0088	0.0109	0.0127
	*rRMSE*	10.27%	5.14%	6.41%

**Table 5:** Statistics on the number of pixels with reflectance differences within a specific threshold.

RE/REI	Forest	Crop	Grass	Stat. Range
RE-1 (705 nm)	99.99%	99.69%	99.64%	(−0.03, 0.03)
RE-2 (740 nm)	99.87%	99.46%	99.72%	(−0.03, 0.03)
RE-3 (783 nm)	99.49%	99.50%	98.71%	(−0.03, 0.03)

**Table 6:** Statistics on the number of pixels with index differences within a specific threshold.

REI	Forest	Crop	Grass	Stat. Range
NDREI	99.81%	98.76%	98.72%	(−0.1, 0.1)
CIre	98.23%	97.17%	99.99%	(−1.0, 1.0)
IRECI	97.25%	96.13%	98.37%	(−0.2, 0.2)

## Data Availability

Data are contained within the article.
